# Renal Injury in All-Comers After Transcatheter Aortic Valve Replacement: A Systematic Review and Meta-Analysis

**DOI:** 10.7759/cureus.7985

**Published:** 2020-05-06

**Authors:** Waqas J Siddiqui, Murrium I Sadaf, Muhammad Zain, Rabia Mazhar, Ramla Abbas, Mohammad H Khan, Faiza Ahmed, Omer Zuberi, Youssef M Al-Saghir, Jesse Goldman, Sandeep Aggarwal

**Affiliations:** 1 Cardiology/Nephrology, Drexel University College of Medicine, Philadelphia, USA; 2 Cardiology/Nephrology, Orange Park Medical Center, Orange Park, USA; 3 Internal Medicine, Yale School of Medicine, New Haven, USA; 4 Internal Medicine, Waterbury Hospital, Waterbury, USA; 5 Internal Medicine, Sheikh Zayed Medical College and Hospital, Rahim Yar Khan, PAK; 6 Internal Medicine, Orange Park Medical Center, Orange Park, USA; 7 Medicine, Wynford Chelation Center, Toronto, CAN; 8 Cardiology, Orange Park Medical Center, Orange Park, USA; 9 Nephrology, Thomas Jefferson University, Philadelphia, USA; 10 Nephrology, University of Pennsylvania, Philadelphia, USA

**Keywords:** transcatheter aortic valve replacement (tavr), savr, surgical aortic valve replacement, acute kidney injury (aki), renal failure, renal transplant, aortic stenosis

## Abstract

Background

Acute kidney injury (AKI) following aortic valve replacement is associated with poor prognosis. Transcatheter aortic valve replacement (TAVR) is a novel strategy with a percutaneous approach and early recovery time. We conducted this meta-analysis to compare TAVR to surgical aortic valve replacement (SAVR) and their respective renal outcomes.

Methods

We searched for randomized controlled trials (RCTs) using MEDLINE, PUBMED, and Google Scholar databases from their inception till April 6, 2019, and included eight trials comparing TAVR to SAVR in cases that reported AKIs.

Results

We found a significant reduction in AKI after TAVR compared to SAVR at 30 days [n = 66 vs. n = 160, respectively; odds ratio (OR) = 0.38, 95% confidence interval (CI) = 0.28-0.51; p: <0.00001, I^2^ = 0%]. At one year, a trend towards reduced renal failure was noted in the TAVR arm compared to the SAVR arm (n = 74 vs. n = 129, respectively; OR = 0.57, 95% CI = 0.32-1.01; p = 0.05, I^2^ = 69%).

Conclusion

Based on our findings and analysis, we have concluded that TAVR is associated with significantly reduced renal injury at 30 days when compared to SAVR.

## Introduction

Severe aortic stenosis (AS) is associated with significant functional impairment in the elderly population with poor prognosis [1]. Only one-third of symptomatic adults with severe AS can be candidates for high-risk surgical interventions due to their underlying comorbidities [2]. Therefore, in recent years, the use of transcatheter aortic valve replacement (TAVR) has become standard of care, with an increase in the number of implants per million adults from 24.8 in 2012 to 63.2 in 2014 compared to surgical aortic valve replacement (SAVR) in intermediate and high surgical risk populations [3-6]. This trend is primarily due to a decreased risk of mortality with TAVR compared to SAVR and a significantly lower risk of acute kidney injury (AKI) with TAVR compared to SAVR [7,8]. Two recently published non-inferiority trials comparing TAVR to SAVR in the low-risk patient population also reported significantly reduced events of AKI in the TAVR arm [7,9]. We recently published a meta-analysis comparing TAVR with SAVR in patients with severe AS and reported their renal outcomes [10]. This is an updated meta-analysis of all the available randomized controlled trials (RCTs) to report AKI at 30 days and one year, and AKI requiring renal transplant in low-, intermediate-, and high-risk candidates. The results of this analysis were also presented at the American Heart Association meeting in 2019 (Paper presentation: Siddiqui WJ, Mazhar R, Abbas R, Sadaf M, Zain M, Omer Z, Al-Saghir Y. Abstract 15718: Acute Kidney Injury After Transcatheter Aortic Valve Replacement vs. Surgical Aortic Valve Replacement - A Meta-Analysis. Meeting of the American Heart Association; 2019).

## Materials and methods

Data sources and search strategy

We conducted our systematic review following the Preferred Reporting Items for Systematic Review and Meta-Analyses (PRISMA) guidelines [11]. We searched for RCTs using MEDLINE, PUBMED, and Google Scholar databases comparing TAVR to SAVR for the treatment of AS using following keywords and MeSH terms: “aortic stenosis, surgical aortic valve replacement, transcatheter aortic valve replacement, transcatheter aortic valve implantation, AS, SAVR, TAVR, and TAVI” from inception to April 6, 2019. Our search strategy included (aortic stenosis) OR (AS) AND (SAVR) OR (surgical aortic valve replacement) AND (TAVR) OR (transcatheter aortic valve replacement) OR (TAVI) OR (transcatheter aortic valve implantation). We used Boolean Operators “AND” and “OR” to combine the search terms. After identifying duplications, a total of 291 studies were finally identified.

Study selection

**Figure 1:**
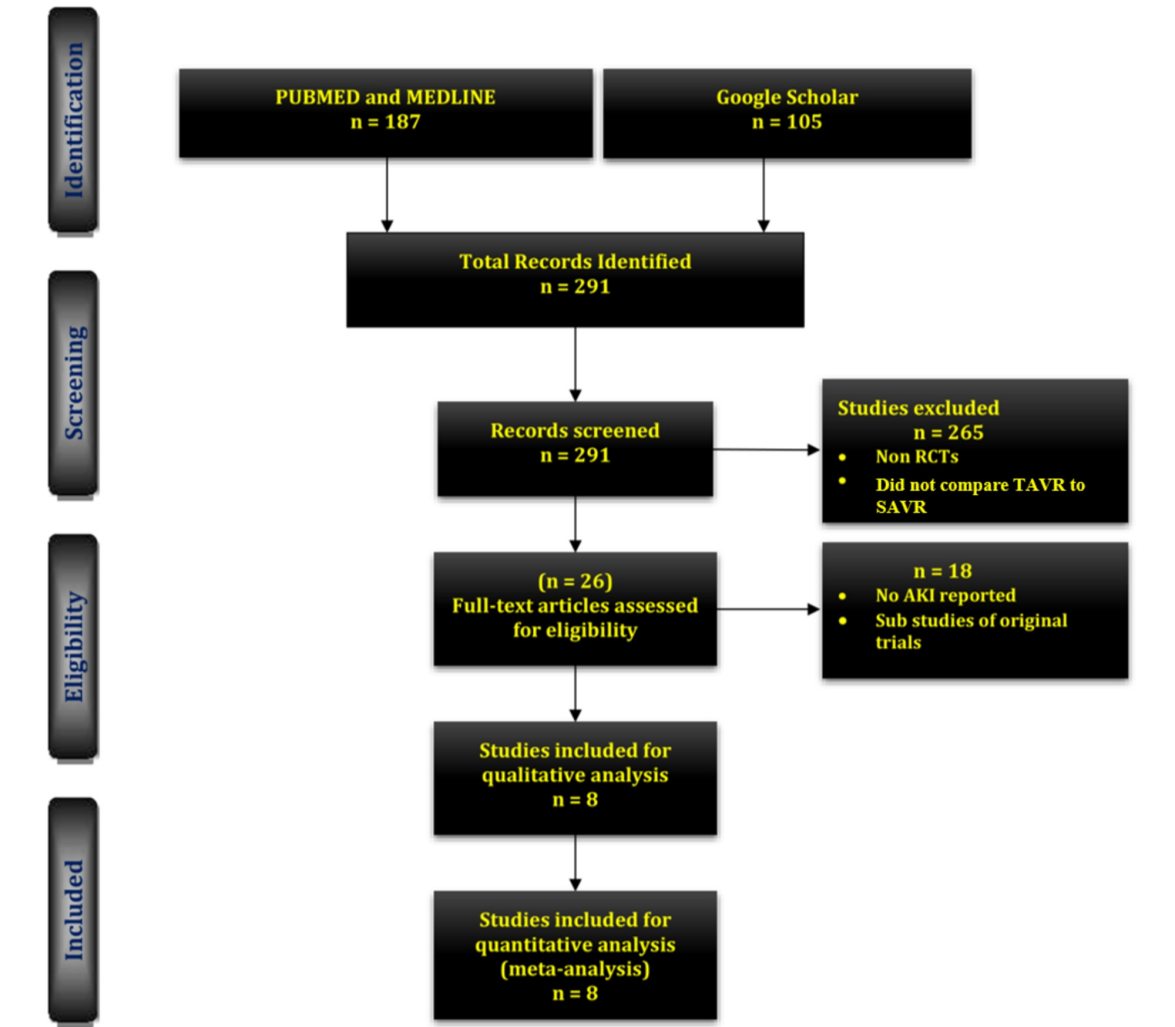
PRISMA diagram AKI: acute kidney injury; TAVR: transcatheter aortic valve replacement; SAVR: surgical aortic valve replacement; RCT: randomized controlled trial; PRISMA: Preferred Reporting Items for Systematic Review and Meta-Analyses

Three reviewers (W.J.S., R.M., and R.A.) reviewed the titles and abstracts, and they excluded 265 studies that failed to meet our inclusion criteria. We assessed the full text of 26 studies; 18 were excluded as they lacked the primary outcome of interest, or since they were sub-studies of original trials. We included eight RCTs for our systematic review and meta-analysis, which compared TAVR outcomes to SAVR outcomes (Figure 1).

Inclusion criteria

The inclusion criteria were as follows: the study should be a prospective RCT comparing TAVR to SAVR for severe AS; patients should be of age ≥18 years; the study sample size must consist of at least 50 patients; AKI should be at least one among the outcomes reported. The primary endpoints we analyzed consisted of AKIs at 30 days, renal injury at one year, and need for renal replacement therapy.

The secondary endpoints we analyzed consisted of all-cause mortality at 30 days and one year, mortality secondary to cardiovascular causes at 30 days and one year, rehospitalizations at 30 days and one year, stroke or transient ischemic attacks at 30 days and one year, incidence of myocardial infarction (MI), postprocedure-related major bleeding, new-onset atrial fibrillation, heart block requiring permanent pacemaker placement, vascular complications, and incidence of valve endocarditis.

Data extraction and quality assessment 

The reviewers W.J.S., R.M., and R.A. extracted data into predefined fields on a Microsoft Excel (Microsoft, Redmond, WA) sheet for baseline characteristics and study outcomes. W.J.S. cross-checked the data and made the necessary corrections. All three reviewers discussed the revisions and reached a consensus on the final entry.

Data synthesis and analysis

Statistical Method

We used the random-effects model and the Mantel-Haenszel method in Review Manager (RevMan) Version 5.3 (The Nordic Cochrane Centre, The Cochrane Collaboration, 2014, Copenhagen) for dichotomous data to calculate the risk and odds ratio (OR) and 95% confidence intervals (CI). We reported results as forest plots. We used GraphPad Online (GraphPad Software, La Jolla, CA) to calculate Chi^2^ to compare the baseline characteristics of two groups. A two-sided p-value of <0.05 was considered statistically significant.

Heterogeneity

We used I^2^ statistics to calculate the heterogeneity. We considered I^2 ^of >50% as substantial heterogeneity, as explained in the Cochrane Handbook for Systematic Reviews [12]. We performed a sensitivity analysis for considerable heterogeneity.

## Results

We included eight RCTs with 7,889 patients (4,017 with TAVR and 3,872 with SAVR) in our analysis. Baseline characteristics and salient features of the studies are summarized in Table 1 [5,6,7,9,13-16]. Primary and secondary outcomes are summarized in Table 2.

**Table 1:** Summary of baseline characteristics and salient features of the studies TAVR: transcatheter aortic valve replacement; SAVR: surgical aortic valve replacement; NEJM: New England Journal of Medicine; JACC: Journal of American College of Cardiology; RCT: randomized controlled trial; N/A: not available; SD: standard deviation

Characteristics	Reardon et al., 2017 [6]		Leon et al., 2016 [5]		Thyregod et al., 2015 [13]		Adams et al., 2014 [14]		Nielsen et al., 2012 [15]		Smith et al., 2011 [16]		Popma et al., 2019 [7]		Mack et al., 2019 [9]	
Journal	NEJM		NEJM		JACC		NEJM		Eurointervention		NEJM		NEJM		NEJM	
Design	Multicenter, prospective RCT		Multicenter, prospective RCT		Multicenter, prospective RCT		Multicenter, prospective RCT		Multicenter, prospective RCT		Multicenter, prospective RCT		Multicenter, prospective RCT		Multicenter, prospective RCT	
Population	Intermediate risk		Low–intermediate risk		Low 82%, intermediate 18% risk		High risk		Low–intermediate risk, requested from ILLiad		High risk for surgery (inoperable)		Low risk for surgery		Low risk for surgery	
	TAVR	SAVR	TAVR	SAVR	TAVR	SAVR	TAVR	SAVR	TAVR	SAVR	TAVR	SAVR	TAVR	SAVR	TAVR	SAVR
	n = 864	n = 796	n = 1,011	n = 1,021	n = 145	n = 135	n = 394	n = 401	n = 34	n = 36	n = 348	n = 351	n = 725	n = 678	n = 496	n = 454
Age, years, mean±SD	79.9±6.2	79.7±6.1	81.5±6.7	81.7±6.7	79.2±4.9	79.0±4.7	83.2±7.1	83.5±6.3	80.0±3.6	82.0±4.4	83.6±6.8	84.5±6.4	74.1±5.8	73.6±5.9	73.3±5.8	73.6±6.1
Male, n (%)	498 (57.6%)	438 (55.0%)	548 (54.2%)	560 (54.8%)	78 (53.8%)	71 (52.6%)	N/A	N/A	9 (26.5%)	12 (33.3%)	201 (57.8%)	198 (56.7%)	464 (64%)	449 (66.2%)	335±67.5	323±71.1
Logistic EuroSCORE, mean±SD	11.9±7.6	11.6±8.0	N/A	N/A	8.4±4.0	8.9±5.5	17.6±13.0	18.4±12.8	9.4±3.9	10.3±5.8	29.3±16.5	29.2±15.6	N/A	N/A	1.5±1.2	1.5±0.9
Diabetes mellitus, n	295	277	381	349	26	28	136	172	1	3	N/A	N/A	228	207	155	137
Hypertension, n	801	719	N/A	N/A	103	103	375	386	N/A	N/A	N/A	N/A	614	559	N/A	N/A
Peripheral vascular disease, n	266	238	282	336	6	9	163	169	2	3	148	142	54	56	34	33
Cerebral vascular disease, n	N/A	N/A	325	317	24	22	N/A	N/A	1	1	95	87	74	80	N/A	N/A
Stroke, n	57	57	N/A	N/A	N/A	N/A	51	53	N/A	N/A	N/A	N/A	N/A	N/A	17	23
Transient ischemic attack, n	58	46	N/A	N/A	N/A	N/A	50	51	N/A	N/A	N/A	N/A	N/A	N/A	N/A	N/A
Coronary artery disease, n	541	511	700	679	N/A	N/A	297	306	N/A	N/A	260	266	N/A	N/A	137	127
Myocardial infarction, n	125	111	185	179	8	6	101	98	N/A	N/A	92	103	48	33	28	26
Coronary artery bypass graft, n	138	137	239	261	N/A	N/A	117	121	N/A	N/A	147	152	18	14	N/A	N/A
Percutaneous coronary intervention, n	184	169	274	282	11	12	133	152	N/A	N/A	116	110	103	87	N/A	N/A
Pacemaker, n	84	72	118	123	5	6	92	83	N/A	N/A	69	76	23	26	12	13
Congestive heart failure, n	824	769	N/A	N/A	N/A	N/A	376	387	N/A	N/A	N/A	N/A	N/A	N/A	N/A	N/A
Balloon valvuloplasty, n (%)	N/A	N/A	51 (5.0%)	50 (4.9%)	N/A	N/A	N/A	N/A	N/A	N/A	46 (13.4%)	35 (10.2%)	N/A	N/A	N/A	N/A
Atrial fibrillation/atrial flutter, n	243	211	313	359	40	34	161	190	N/A	N/A	80	73	111	98	78	85
New York Heart Association Class, n																
Class II	344	333	N/A	N/A	67	70	56	53	N/A	N/A	20	21	467	422	N/A	N/A
Class III	472	411	N/A	N/A	67	57	258	277	N/A	N/A	328	328	181	190	155	108
Class IV	48	52	N/A	N/A	3	4	80	71	N/A	N/A	328	328	1	3		
Society of Thoracic Surgeons Predictive Risk of Mortality mean, ±SD	N/A	N/A	5.8±2.1	5.8±1.9	2.9±1.6	3.1±1.7	7.3±3.0	7.5±3.2	3.1±1.5	3.4±1.2	N/A	N/A	1.9±0.7	1.9±0.7	1.9±0.7	1.9±0.6
Society of Thoracic Surgeons Predictive Risk of Mortality mean, n	N/A	N/A	N/A	N/A	N/A	N/A	48	52	N/A	N/A	N/A	N/A	N/A	N/A	N/A	N/A
Creatinine level of >2 mg/dl (177 µmol/lit), n (%)	14 (1.6%)	17 (2.1%)	51 (5.0%)	53 (5.2%)	2 (1.4%)	1 (0.7%)	N/A	N/A	1 (2.9%)	0	38 (11.1%)	24 (7.0%)	3 (0.4%)	1 (0.1%)	0.2	0.2
Aortic valve area (cm^2^), mean±SD	N/A	N/A	0.7±0.2	0.7±0.2	N/A	N/A	N/A	N/A	0.66±0.17	0.71±0.17	0.7±0.2	0.6±0.2	0.8±0.2	0.8±0.2	0.8±0.2	0.8±0.2
Aortic-valve gradient (mmHg), mean±SD	N/A	N/A	44.9±13.4	44.6±12.5	N/A	N/A	N/A	N/A	81±26	66±23	42.7±14.6	43.5±14.3	47±12.1	46.6±12.2	49.4±12.8	48.3±11.8
Left ventricular ejection fraction (%), mean±SD	N/A	N/A	56.2±10.8	55.3±11.9	N/A	N/A	N/A	N/A	56.5±9.7	56.3±10	52.5±13.5	53.3±12.8	61.7 (7.9%)	61.9 (7.7%)	65.7±9.0	66.2±8.6

**Table 2:** Primary and secondary outcomes TAVR: transcatheter aortic valve replacement; SAVR: surgical aortic valve replacement; CV: cardiovascular; CI: confidence interval

Outcome	TAVR, n	SAVR, n	Effect estimate	95% CI	P-value	I^2^
Primary outcomes						
Acute kidney injury at 30 days	66	160	0.38	0.28–0.51	<0.00001	0%
Acute kidney injury at 1 year	74	129	0.57	0.32–1.01	0.05	69%
Acute kidney injury requiring renal replacement therapy	20	23	0.87	0.47–1.62	0.67	0%
Secondary outcomes						
Cerebral vascular accident or transient ischemic attack at 30 days	179	197	0.91	0.65–1.25	0.55	43%
Cerebral vascular accident or transient ischemic attack at 1 year	266	283	0.94	0.72–1.24	0.68	51%
Major bleeding	496	954	0.46	0.26–0.81	0.008	95%
Major vascular complications	170	61	2.77	1.52–5.06	0.0009	70%
Myocardial infarction	41	44	0.87	0.56–1.34	0.52	0%
Mortality from any cause at 30 days	94	112	0.8	0.59–1.08	0.15	5%
Mortality from any cause at 1 year	349	375	0.89	0.76–1.04	0.15	0%
Mortality from any CV cause at 30 days	71	74	0.94	0.68–1.31	0.73	0%
Mortality from any CV cause at 1 year	180	198	0.88	0.7–1.1	0.25	8%
New-onset atrial fibrillation	343	1,009	0.24	0.16–0.37	<0.00001	89%
Need for permanent pacemaker	555	179	3.03	1.77–5.2	<0.0001	87%
Rehospitalizations at 30 days	66	91	0.67	0.46–0.98	0.04	23%
Rehospitalizations at 1 year	190	198	0.85	0.54–1.33	0.47	78%
Valve endocarditis	8	10	0.77	0.28–2.06	0.6	0%

Primary outcomes

**Figure 2:**
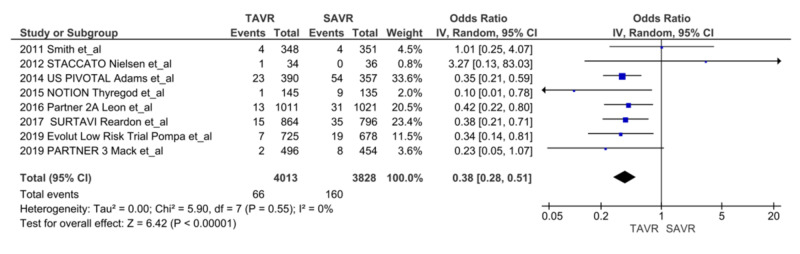
AKI at 30 days AKI: acute kidney injury; TAVR: transcatheter aortic valve replacement; SAVR: surgical aortic valve replacement; CI: confidence interval

**Figure 3:**
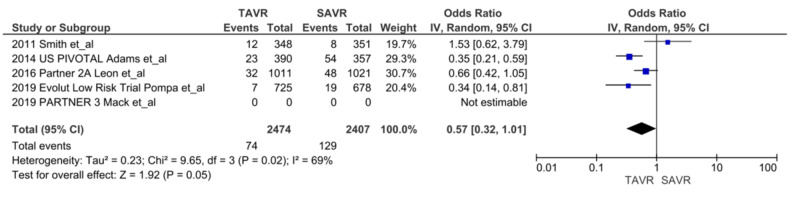
AKI at one year AKI: acute kidney injury; TAVR: transcatheter aortic valve replacement; SAVR: surgical aortic valve replacement; CI: confidence interval

**Figure 4:**
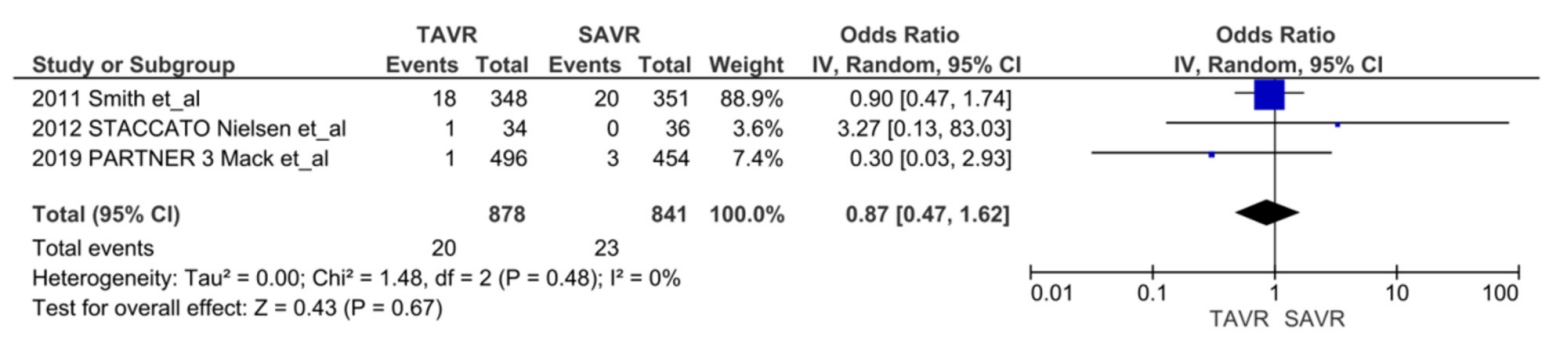
AKI with renal replacement therapy AKI: acute kidney injury; TAVR: transcatheter aortic valve replacement; SAVR: surgical aortic valve replacement; RRT: renal replacement therapy; CI: confidence interval

We noted a significant reduction in AKIs at 30 days after TAVR compared with SAVR, (n = 66 vs. n = 160, respectively; OR = 0.38, 95% CI = 0.28-0.51; p = <0.00001, I^2^ = 0%) (Figure 2) [5,6,7,9,13-16]. We also noted a reduction in the trend of persistent renal injury at one year after TAVR compared to SAVR (n = 74 vs. n = 129, respectively; OR = 0.57, 95% CI = 0.32-1.01; p = 0.05, I^2^ = 69%). With sensitivity analysis without Smith et al., results became significant favoring TAVR (OR = 0.45, CI = 0.28-0.73, p = 0.001, I^2^ = 49%) (Figure 3) [5,7,9,14,16]. There was no difference in the need for renal replacement therapy (RRT) in the TAVR group compared to the SAVR group (n = 20 vs. n = 23, respectively; OR = 0.87, 95% CI = 0.47-1.62, p = 0.67, I^2 ^= 0%) (Figure 4) [9,15,16].

Secondary outcomes

As noted in Table 2, we found no significant differences between TAVR and SAVR in many secondary outcomes with several notable exceptions. TAVR yielded significantly reduced rehospitalizations at 30 days compared with SAVR (n = 66 vs. n = 91, respectively; OR = 0.67, 95% CI = 0.46-0.98; p = 0.04, I^2^ = 23%). The TAVR approach had significantly reduced postprocedure-related major bleeding compared with the SAVR approach (n = 496 vs. n = 954, respectively; OR = 0.46, 95% CI = 0.26-0.81; p = 0.008, I^2^ = 95%) and incidence of new-onset atrial fibrillation (n = 343 vs. n = 1009, respectively; OR = 0.24, 95% CI = 0.16-0.37; p = <0.00001, I^2^ = 89%). TAVR patients required more permanent pacemaker placement than SAVR patients (n = 555 vs. n = 179, respectively; OR = 3.03, 95% CI = 1.77-5.2; p = <0.0001, I^2^ = 87%) and had a higher incidence of vascular complications (n = 170 vs. n = 61, respectively; OR = 2.77, 95% CI = 1.52-5.06, p = 0.0009, I^2^ = 70%).

## Discussion

AKI remains a significant concern following TAVR and SAVR. Our meta-analysis showed that the patients who underwent TAVR had significantly better renal outcomes at 30 days compared to patients who underwent SAVR. However, we observed no difference in TAVR or SAVR in terms of the persistent renal injury and need for RRT at one year. One study showed that improvement in stroke volume and cardiac output after SAVR or TAVR increases the level of renal perfusion, which itself favors the improved renal function and supports our study’s results. However, the study further claims that patients with baseline chronic kidney disease (CKD) are at increased risk of persistent renal injury and need RRT after aortic valve replacement [17]. One retrospective study concluded that as the estimated glomerular filtration rate drops below 30 ml/min/m2, the need for RRT is increased to one in six patients with mortality increasing to one in three in CKD-4 patients at one year [18]. A study done in the UK about the post-TAVR need for RRT has suggested that the requirement for RRT depends not only on baseline renal function but a compromised left ventricular function, history of diabetes mellitus, post-procedural paravalvular leakage, type of valve used, and route of peripheral access other than transfemoral access during TAVR [19]. We also know that the use of cardiopulmonary bypass with extracorporeal circulation during SAVR is a risk factor for patients with AKI or long-standing CKD, and only three RCTs had persistent renal failure at one year, and two RCTs had the need for RRT at the one-year interval. Hence, we think that these results are biased as the data regarding the potential cases with the possibility of developing CKD and requiring RRT after a year of AVR are unavailable. Therefore, more data from RCTs documenting the baseline renal function as well as data from the renal standpoint at a one-year interval are mandatory to evaluate this trend better.

The data regarding the specific intervention to prevent AKI following TAVR are also scarce. It was interesting to see the results from the Prevention of Serious Adverse Events Following Angiography (PRESERVE) trial, which enrolled over 5,000 patients with CKD of 3b or worse without diabetes, or those with CKD of 3a or worse with diabetes. Patients underwent either a coronary or noncoronary angiogram. The administration of normal saline, sodium bicarbonate, or acetylcysteine over placebo failed to demonstrate any benefit [20]. As the contrast administration is the primary risk factor associated with AKI following TAVR, especially in patients with underlying CKD, Chatani et al. found no advantage of using iso-osmolar contrast agent compared with a low-osmolar agent during TAVI for preventing post-TAVR AKI [21]. Barbanti et al.’s trial is the only trial done in patients undergoing TAVR to demonstrate the benefit of forced diuresis using the RenalGuard system (RenalGuard Solutions, Inc., Milford, MA) with furosemide and saline over standard saline alone. The forced diuresis technique can be considered in future RCTs, with the principal focus on patients with underlying CKD [22].

In our study, there was no significant difference between the TAVR and SAVR subgroups in terms of risk of stroke, transient ischemic attack, MI, and mortality from any cause as well as cardiovascular causes at 30 days and one year. However, we saw a reduction in the incidence of major bleeding, major vascular complications, rehospitalizations at 30 days, and new-onset atrial fibrillation in the TAVI group. Although studies have consistently shown a lower incidence of new-onset atrial fibrillation in patients who underwent TAVR compared with SAVR, the rate of permanence remains unknown. Amat-Santos et al. found that the incidence of new-onset atrial fibrillation and stroke increased following TAVR with large atrial size and when opting for the transapical TAVR route [23]. Tarantini et al. reported an association of post-TAVR new-onset atrial fibrillation with a higher incidence of stroke rates at long-term follow-up [24]. A study by Holmes et al., which studied data from the TAVR therapies registry, reported the incidence of new-onset atrial fibrillation following TAVR to be 6.3% [25]. The incidence of permanent pacemaker (PPM) installation rates was common after TAVR. Conduction disturbances requiring PPM following TAVR are a known complication and appear to be unrelated to valve type [26-28]. Factors of post-TAVR PPM implantation incidence can be predicted by pre-existing right bundle branch block (RBBB), the prosthesis to left ventricular outflow tract diameter ratio, and the left ventricular end-diastolic diameter with a longer duration of hospital stay.

From our review and analysis, we propose an algorithm for deciding between TAVR or SAVR in patients with severe AS according to baseline CKD and electrocardiographic (EKG) changes (presented in Figure 5). It is imperative to differentiate patients by surgical risk, especially those with high-risk from low-to-intermediate surgical risk patients. For patients with low-to-intermediate risk for surgery, the decision regarding TAVR or SAVR should be left to the patients themselves; however, for high surgical risk, the physician should consider TAVR along with two additional considerations. First, if the patient has underlying baseline EKG abnormalities such as heart block or RBBB, a physician should consider electrophysiology consult before TAVR for a preemptive placement of a PPM. Second, if the patient has no CKD or has CKD-1 to CKD-3, the physician should proceed with TAVR. In patients with advanced CKD-4, CKD-5, or in those already on hemodialysis, physicians should discuss the benefits and adverse outcomes of TAVR with the patient, such as worse hospital outcomes and higher incidence of mortality. The physician should also offer the patient SAVR as an alternative option.

Our analysis has several limitations. First, we included all RCTs that randomized patients with low, intermediate, and high surgical risk for SAVR. Secondly, most patients who were recruited had a stable renal function without advanced CKD. The included trials used different valves for TAVR, which makes it challenging to identify if one valve has any protective role in renal outcomes over another. Also, renal outcomes addressed by each trial were either secondary outcomes or were reported in the supplementary appendix. Finally, we had no access to the patient-level data to identify baseline renal function.

## Conclusions

Our analysis showed that TAVR is associated with a significant reduction in renal injury in patients compared to SAVR. Given the success associated with TAVR, novel and robust measures are needed to minimize the renal injury that is associated with poor outcomes post valve replacement.

## References

[1] Carabello BA, Paulus WJ (2009). Aortic stenosis. Lancet.

[2] Bach DS, Siao D, Girard SE, Duvernoy C, McCallister BD Jr, Gualano SK (2009). Evaluation of patients with severe symptomatic aortic stenosis who do not undergo aortic valve replacement: the potential role of subjectively overestimated operative risk. Circ Cardiovasc Qual Outcomes.

[3] Nishimura RA, Otto CM, Bonow RO (2017). 2017 AHA/ACC focused update of the 2014 AHA/ACC guideline for the management of patients with valvular heart disease: a report of the American College of Cardiology/American Heart Association Task Force on Clinical Practice Guidelines. Circulation.

[4] Gupta T, Kalra A, Kolte D (2017). Regional variation in utilization, in-hospital mortality, and health-care resource use of transcatheter aortic valve implantation in the United States. Am J Cardiol.

[5] Leon MB, Smith CR, Mack MJ (2016). Transcatheter or surgical aortic-valve replacement in intermediate-risk patients. N Engl J Med.

[6] Reardon MJ, Van Mieghem NM, Popma JJ (2017). Surgical or transcatheter aortic-valve replacement in intermediate-risk patients. N Engl J Med.

[7] Popma JJ, Deeb GM, Yakubov SJ (2019). Transcatheter aortic-valve replacement with a self-expanding valve in low-risk patients. N Engl J Med.

[8] Himbert D, Descoutures F, Al-Attar N (2009). Results of transfemoral or transapical aortic valve implantation following a uniform assessment in high-risk patients with aortic stenosis. J Am Coll Cardiol.

[9] Mack MJ, Leon MB, Thourani VH (2019). Transcatheter aortic-valve replacement with a balloon-expandable valve in low-risk patients. N Engl J Med.

[10] Siddiqui WJ, Alvarez C, Aslam M (2018). Meta-analysis comparing outcomes and need for renal replacement therapy of transcatheter aortic valve implantation versus surgical aortic valve replacement. Am J Cardiol.

[11] Liberati A, Altman DG, Tetzlaff J (2009). The PRISMA statement for reporting systematic reviews and meta-analyses of studies that evaluate healthcare interventions: explanation and elaboration. BMJ.

[12] Higgins JP, Altman DG, Gøtzsche PC (2011). The Cochrane Collaboration's tool for assessing risk of bias in randomised trials. BMJ.

[13] Thyregod HG, Steinbrüchel DA, Ihlemann N (2015). Transcatheter versus surgical aortic valve replacement in patients with severe aortic valve stenosis: 1-year results from the all-comers NOTION randomized clinical trial. J Am Coll Cardiol.

[14] Adams DH, Popma JJ, Reardon MJ (2014). Transcatheter aortic-valve replacement with a self-expanding prosthesis. N Engl J Med.

[15] Nielsen HH, Klaaborg KE, Nissen H (2012). A prospective, randomised trial of transapical transcatheter aortic valve implantation vs. surgical aortic valve replacement in operable elderly patients with aortic stenosis: the STACCATO trial. EuroIntervention.

[16] Smith CR, Leon MB, Mack MJ (2011). Transcatheter versus surgical aortic-valve replacement in high-risk patients. N Engl J Med.

[17] Xu JR, Zhuang YM, Liu L (2017). Reversible preoperative renal dysfunction does not add to the risk of postoperative acute kidney injury after cardiac valve surgery. Ther Clin Risk Manag.

[18] Hansen JW, Foy A, Yadav P (2017). Death and dialysis after transcatheter aortic valve replacement: an analysis of the STS/ACC TVT registry. JACC Cardiovasc Interv.

[19] Ferro CJ, Law JP, Doshi SN (2017). Dialysis following transcatheter aortic valve replacement: risk factors and outcomes: an analysis from the UK TAVI (Transcatheter Aortic Valve Implantation) registry. JACC Cardiovasc Interv.

[20] Weisbord SD, Gallagher M, Jneid H (2018). Outcomes after angiography with sodium bicarbonate and acetylcysteine. N Engl J Med.

[21] Chatani K, Abdel-Wahab M, Wübken-Kleinfeld N (2015). Acute kidney injury after transcatheter aortic valve implantation: impact of contrast agents, predictive factors, and prognostic importance in 203 patients with long-term follow-up. J Cardiol.

[22] Barbanti M, Gulino S, Capranzano P (2015). Acute kidney injury with the RenalGuard System in patients undergoing transcatheter aortic valve replacement: the PROTECT-TAVI Trial (PROphylactic effecT of furosEmide-induCed diuresis with matched isotonic intravenous hydraTion in Transcatheter Aortic Valve Implantation). JACC Cardiovasc Interv.

[23] Amat-Santos IJ, Rodés-Cabau J, Urena M (2012). Incidence, predictive factors, and prognostic value of new-onset atrial fibrillation following transcatheter aortic valve implantation. J Am Coll Cardiol.

[24] Tarantini G, Mojoli M, Windecker S (2016). Prevalence and impact of atrial fibrillation in patients with severe aortic stenosis undergoing transcatheter aortic valve replacement: an analysis from the SOURCE XT prospective multicenter registry. JACC Cardiovasc Interv.

[25] Holmes DR Jr, Brennan JM, Rumsfeld JS (2015). Clinical outcomes at 1 year following transcatheter aortic valve replacement. JAMA.

[26] Gargiulo G, Sannino A, Capodanno D (2016). Transcatheter aortic valve implantation versus surgical aortic valve replacement: a systematic review and meta-analysis. Ann Intern Med.

[27] Siemieniuk RA, Agoritsas T, Manja V (2016). Transcatheter versus surgical aortic valve replacement in patients with severe aortic stenosis at low and intermediate risk: systematic review and meta-analysis. BMJ.

[28] Siontis GC, Praz F, Pilgrim T (2016). Transcatheter aortic valve implantation vs. surgical aortic valve replacement for treatment of severe aortic stenosis: a meta-analysis of randomized trials. Eur Heart J.

